# Maternal Mortality in Brazil, 1990 to 2019: a systematic analysis of the Global Burden of Disease Study 2019

**DOI:** 10.1590/0037-8682-0279-2021

**Published:** 2022-01-28

**Authors:** Lisiane Freitas Leal, Deborah Carvalho Malta, Maria de Fatima Marinho Souza, Ana Maria Nogales Vasconcelos, Renato Azeredo Teixeira, Guilherme Augusto Veloso, Sônia Lansky, Antonio Luiz Pinho Ribeiro, Giovanny Vinícius Araújo de França, Mohsen Naghavi

**Affiliations:** 1 McGill University, Department of Epidemiology, Biostatistics and Occupational Health, Montreal, QC, Canada.; 2 Universidade Federal de Minas Gerais, Escola de Enfermagem, Departamento de Enfermagem Materno-Infantil e Saúde Pública, Belo Horizonte, MG, Brasil.; 3 Ministério da Saúde, Secretaria de Vigilância em Saúde, Departamento de Análise da Situação de Saúde, Brasília, DF, Brasil.; 4 Universidade de Brasília - Campus Darcy Ribeiro, Departamento de Estatística, Instituto de Ciências Exatas, Brasília, DF, Brasil.; 5 Universidade Federal de Minas Gerais, Faculdade de Medicina, Programa de Pós-Graduação em Saúde Pública, Belo Horizonte, MG, Brasil.; 6 Universidade Federal de Minas Gerais, Departamento de Estatística, Programa de Pós-graduação em Estatística, Belo Horizonte, MG, Brasil.; 7 Secretaria Municipal de Saúde Belo Horizonte, Belo Horizonte, MG, Brasil.; 8 Universidade Federal de Minas Gerais, Faculdade de Medicina, Departamento de Clínica Médica, Belo Horizonte, MG, Brasil.; 9 Ministério da Saúde, Secretaria de Vigilância em Saúde, Brasília, DF, Brasil.; 10 Institute for Health Metrics and Evaluation, Seattle, WA, United States of America.

**Keywords:** Maternal mortality, Cause of death, Global Burden of diseases, World Health Organization, Brazil

## Abstract

**INTRODUCTION:**

Maternal death continues to be one of the most challenging public health problems that needs to be addressed in low and middle-income countries. The objective of this study was to describe the problem of maternal death in Brazil, using estimates from the Global Burden of Disease Study (GBD).

**METHODS:**

This study used data from the GBD 2019 to show the numbers of deaths and the Maternal Mortality Ratio (MMR) - number of deaths/100,000 live births - in Brazil and its 27 Federated Units (FU), for ages 10 to 54 years, from 1990 to 2019. The annual variation of the MMR was estimated in 1990, 2010, and 2019. The MMR were shown for specific causes as well as for five-year age groups. The estimates were presented with 95% uncertainty intervals (UI).

**RESULTS:**

The number of maternal deaths, as well as the MMR showed a 49% reduction from 1990 to 2019. This reduction occurred heterogeneously throughout the country, and the profile of the MMR for specific causes changed between 1990 and 2019: from hypertensive gestation diseases, to indirect maternal deaths, followed by hypertensive gestation diseases. In the extreme age groups, the MMR is higher, with mortality increasing exponentially in direct proportion with age.

**CONCLUSIONS:**

Maternal deaths in Brazil have decreased substantially since 1990; however, the numbers still fall short of what was established by the World Health Organization (WHO). Indirect causes are the greatest problem in more than 60% of the FU, especially for hypertensive pregnancy diseases.

## INTRODUCTION

Maternal death, defined as the death of women during gestation, abortion, delivery, and post-delivery - due to any cause related or aggravated by pregnancy, regardless of its duration - or the period which extends for 42 days after its end^1^, continues to be one of the most complex public health problems and one of the most difficult to solve in the low or medium-income countries^2^.

The World Health Organization (WHO) divides maternal death into direct causes (complications related to interventions, omissions, incorrect treatment due to the obstetric state), and indirect causes (resulting from existing diseases or diseases which developed during pregnancy), as well as the definitions of late maternal death (death of a woman by indirect or direct causes more than 42 days after delivery, but within less than a year since interruption of the pregnancy)^1^. The importance of classifying deaths as direct or indirect has been discussed based on the argument that, regardless of etiology, maternal deaths continue to occur without being identified, and when they are identified, they are not classified correctly; therefore, the objective of directing interventions and prevention is not met^3^.

The WHO, subsidized by the Work Group for Classification of Maternal Mortality and Morbidity, established the standards to identify maternal deaths, using the 10th Revision of the International Classification of Diseases (ICD-10). The document seeks to standardize the identification of causes of death, to ensure their registration in a consistent manner, and to improve the reliability of the measurements established by the Millennium Development Goals (MDGs)^4^ and subsequently by the Sustainable Development Goals (SDGs)^5^.

Goal 5 of the MDG was a 75% reduction in MMR between 1990 and 2015^4^ ^,^ ^6^. The MMR is the indicator that demonstrates the ratio of the number of maternal deaths, using as denominator 100,000 live births^7^, and its values subsidize decision-making related to providing adequate health services, from family planning to assistance in delivery and puerperium^8^.

This ambitious goal was not met by the majority of the countries^9^, among them Brazil, which should reach an MMR of 35.8^10^. With the end of the MDG in 2015, and the establishment of the SDG^11^, new targets for health improvement were established, among those, reaching less than 70 maternal deaths for 100,000 live births globally until 2030^12^. Brazil established its target to reach an MMR of 30^13^, which will require considerable efforts in the coming years.

Regardless of the efforts and improvements in the quality of records and in the coverage of inquiries on maternal deaths over the years, there is still heterogeneity in terms of surveillance among Brazilian states^14^. The MMR, at the national and state levels, is calculated by the Ministry of Health, using correction factors, considering the existence of underreporting and failures in the process of investigation of maternal deaths. Szwarcwald et al.^15^ demonstrated that not only the underreporting of deaths must be considered, but also the underreporting of the live births, which is made available by the system of Information on the Live Births (SINASC, in Portuguese) and has an impact on the estimates of MMR.

These corrections, however, make the Brazilian estimates not comparable to those from other countries^6^. However, the GBD estimates enable comparability, since they are based on standardized methods, which are updated annually.

Therefore, this study aimed to describe maternal mortality in Brazil between 1990 and 2019, using data from the GBD.

## METHODS

This study is based on the mortality estimates from the GBD 2019 study. The results of the GBD 2019 are available through an interactive data downloading tool from the Global Health Data Exchange (GHDx). The online version of the GBD 2019 is available at http://vizhub.healthdata.org/GBD-compare, and provides results for all the estimated measurements and nearly all the countries around the world. The GBD 2019 synthesized a large, growing number of data entry sources, including studies, census, vital statistics, and other sources of data related to health. For Brazil, besides the national estimate, it is possible to do separate analyses for its 27 Federated Units (26 States and the Federal District).

The GBD 2017 Causes of Death Collaborators published the detailed process of modelling of individual causes of mortality^16^ and updated procedures for estimating maternal death used by the GBD 2019 were published as well^17^. Briefly, all the data available on causes of death (CoD) is standardized and grouped in a single dataset used to generate mortality estimates by specific causes, for different ages, gender, year, and location. There are 14 steps until the final MMR estimates are obtained, and seven types of data are used to create the CoD bank: vital records (VR), verbal autopsies (VA), cancer records, police records, narrative from siblings, surveillance, survey/census, and diagnoses of samples of invasive tissue. For the construction of the 1990-2019 estimates for Brazil, the GBD used 126 different sources of data for the mortality estimates and 51 for the estimates of causes of death, using the Mortality Information System (SIM, in Portuguese) as its main source (Supplementary material Tables 1S **and** 2S). 

Once the sources of data have been standardized, the mapping of the codes of the ICD was performed, and the “Garbage Codes” (non-specific codes) were redistributed. Data on deaths caused by HIV are analyzed separately, including the databank of only maternal deaths by HIV (incidental deaths, not maternal, are excluded). Bayesian Models are applied to reduce problems related to the noise that might be generated due to counting, which includes zero, thus generating the base of the CoD.

Distinctive modelling strategies are used to estimate maternal mortality in general and mortality by specific causes. For maternal mortality in general, the modelling of the causes of death (CODEm)^18^, is applied, including a group of previously specified covariates (Supplementary material Table 3S). Mortality by specific causes is derived by the multiplication of two components. The first comprises the proportions predicted by a meta-regression Bayesian tool (DisMod-MR 2.1) designed as a geographic cascade. Worldwide data are modelled by producing initial estimates for predictor variables and the adjustments for alternative characteristics of the study. The second is the total number of maternal deaths for that age group, location, and year. In this process, Models of Space-Time Gaussian Regression (ST-GPR) are used in order to capture spatiotemporal patterns^17^. All the MMR estimates are done for each of the eight maternal causes: (1) Maternal deaths aggravated by HIV/AIDS; (2) Hemorrhage; (3) Maternal Sepsis and other maternal infection; (4) Obstructed labor and uterus rupture; (5) Abortion, spontaneous abortion, and ectopic pregnancy; (6) Maternal hypertension; (7) Other direct maternal problems; and (8) Indirect maternal deaths. For each of the specific causes, a different group of covariates is used in order to generate the final estimates (Supplementary material Table 4S). Those causes are included in the GBD analysis if they occur during pregnancy or within a year after the end of pregnancy for women of 10 to 54 years of age. The definitions and the ICD-9 and ICD-10 codes used for each of the causes is demonstrated in Supplementary material Tables 5S **and** 6S.

In this article, the numbers of deaths and MMR are presented, estimated as the number of deaths per 100,000 live births for Brazil and for each of the 27 Federated Units (FU), for ages 10 to 54 years, from 1990 to 2019. For the estimation of the MMR, the database of live births was made available by the Institute for Health Metrics and Evaluation (IHME). The annual variation of MMR was estimated for Brazil and for each FU, in 1990, 2010, and 2019. The MMR was presented for specific causes and five-year age groups. All of the estimates are presented with 95% uncertainty intervals (UI). The 95% UI were estimated by GBD 2019. They are derived from 1,000 samples extracted from the posterior distribution in the estimation process. These limits are distinct from confidence intervals that capture the uncertainty associated with sampling error only. The UIs provide a method of propagating uncertainty from multiple sources, including sampling, estimation, and model specification. The UIs represent the 2.5% and 97.5% percentiles of the 1,000 samples that are determined using the standard CODEm (cause of death ensemble modelling) methodology^16^.

GBD analysis are conducted in R and Python. For this work, figures were produced using the package ggplot2 in RStudio^19^.

The project of the GBD-Brazil study was approved by the Research Ethics Committee of the Universidade Federal de Minas Gerais (CAAE Project Number 62803316.7.0000.5149).

## RESULTS

**FIGURE 1:**
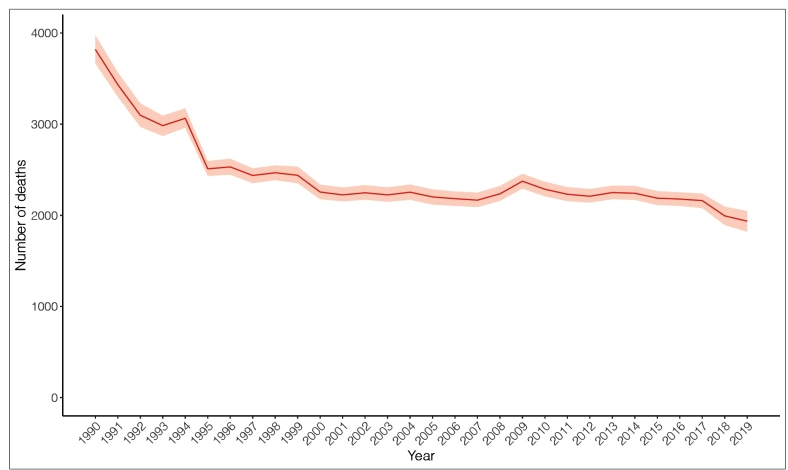
Number of maternal deaths in women aged 10 to 54 years, with an uncertainty interval of 95%, Brazil, 1990-2019.

The number of maternal deaths showed an important reduction between 1990 and 2019 and a global decline of approximately 39% can be estimated over the last 30 years: from 3,818 deaths (95% UI:3,664-3,974) in 1990 to 1,935 (95% UI: 1,818-2,047) in 2019 (Figure 1).

**FIGURE 2:**
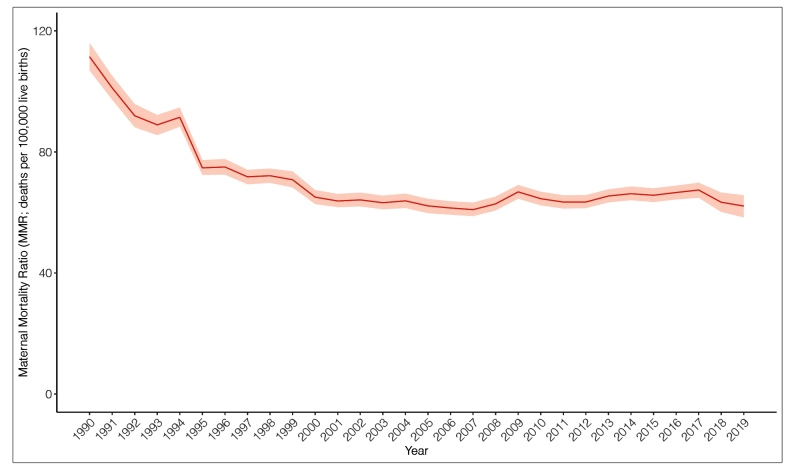
Maternal mortality ratio (per 100,000 live births), age-standardized between 10 and 54 years, with an uncertainty interval of 95%, Brazil, 1990-2019.

In Figure 2, a reduction in MMR was also noticed; the estimate for 1990 was practically twice as much as the estimate for 2019. During this period, a decline in MMR was found from 111.4 deaths per 100,000 live births (95% UI: 106.9-116.0) to 62.1 deaths per 100,000 live births (95% UI: 58.4-65.7). The sharpest reductions occurred mostly from 1990 to 2001, a decrease of 43%. After 2001, the MMR remained practically stable, with oscillations over time. An increase of 6% was observed between 2008 and 2009. The lowest MMR was in 2007 (60.9 deaths per 100,000 live births; 95% UI: 58.8-63.3).

Table 1 shows the MMR for the FU in 1990, 2010, and 2019, and the annual percentage variation. Among the 27 FU, only 14 states showed consistent reductions in MMR. Of those, only 5 presented reductions of more than 50% in MMR. Pernambuco showed the highest reduction, followed by Bahia, Rio Grande do Norte, Minas Gerais, and Alagoas.

**TABLE 1:** Maternal mortality ratio (MMR; number of deaths per 100,000 live births) and annualized percentage change rates, Brazil, 1990, 2010, and 2019.

Regions/Federal Units	Maternal mortality ratio, 95% uncertainty interval (UI)			Percentage of change, 1990-2019
	1990	2010	2019	
Brazil	111.4 (106.9-116.0)	64.6 (62.3-66.9)	62.1 (58.4-65.7)	-44.3 (-48.1; -40.3)
**North**				
Acre	67.7 (57.4-77.8)	66.1 (60.2-72.0)	56.9 (48.2-65.6)	-16 (-32.1; 3.1)
Amapá	74.5 (58.5-88.0)	62.6 (55.4-70.3)	81.0 (69.8-94.2)	8.7 (-13.8; 43.3)
Amazonas	117.1 (96.2-137.1)	90.2 (79.4-102.7)	84.2 (69.8-101.4)	-28.1 (-43.6; -7.7)
Pará	125.1 (102.6-149.7)	71.3 (60.1-84.7)	75.2 (63.2-89.3)	-39.9 (-53; -21.9)
Rondônia	89.2 (63.2-112.8)	80.4 (70.9-89.5)	76.3 (62.8-91.4)	-14.4 (-37.9; 29.4)
Roraima	47.2 (34.5-59.2)	40.4 (35.3-46.0)	45.7 (39.2-53.0)	-3 (-25.9; 35.5)
Tocantins	68.2 (54.6-82.0)	64.5 (54.4-74.7)	68.1 (54.3-84.1)	-0.1 (-23.4; 30.5)
**Northeast**				
Alagoas	115.2 (102.0-130.2)	56.3 (50.2-62.9)	51.9 (42.0-62.8)	-55 (-64.7; -43.6)
Bahia	190.2 (161.3-220.3)	71.4 (60.5-84.7)	64.6 (49.9-82.3)	-66 (-74.7; -54.8)
Ceará	52.9 (42.0-65.1)	61.7 (50.7-75.0)	54.5 (41.6-70.4)	3 (-26.4; 47.0)
Maranhão	78.8 (60.5-99.8)	58.6 (46.0-74.0)	58.0 (43.3-74.4)	-26.4 (-49.6; 7.3)
Paraíba	53.2 (46.5-60.1)	47.7 (42.7-53.1)	49.8 (40.9-60.5)	-6.3 (-27.5; 17.8)
Pernambuco	223.1 (208.1-238.2)	56.4 (51.8-61.3)	56.7 (46.9-67.3)	-74.6 (-79.3; -69.2)
Piauí	84.9 (72.9-98.9)	73.8 (66.6-80.8)	68.5 (56.3-81.2)	-19.4 (-36.8; 0.8)
Rio Grande do Norte	101.0 (84.9-118.9)	39.9 (33.5-47.1)	43.7 (34.0-55.9)	-56.7 (-67.8; -42.7)
Sergipe	93.3 (80.1-106.5)	73.2 (64.1-84.3)	72.9 (57.1-90.5)	-21.8 (-40.5; 3.3)
**South-East**				
Espírito Santo	79.3 (72.5-86.4)	63.7 (56.6-70.6)	66.5 (53.6-80.6)	-16.2 (-33.1; 4.0)
Minas Gerais	118.7 (108.8-129.8)	51.5 (46.5-56.9)	52.4 (43.9-62.9)	-55.8 (-63.8; -47.1)
Rio de Janeiro	109.5 (103.0-116.1)	103.0 (97.7-108.5)	88.2 (74.0-103.8)	-19.4 (-32.7; -3.8)
São Paulo	91.3 (83.5-99.5)	55.7 (52.5-59.2)	59.8 (50.6-70.9)	-34.5 (-46.1; -20.5)
**South**				
Paraná	112.4 (106.0-118.9)	69.2 (64.1-74.4)	60.3 (49.2-72.5)	-46.4 (-56.2; -34.9)
Rio Grande do Sul	110.9 (102.7-119.0)	89.9 (83.4-97.0)	66.4 (54.7-80.8)	-40.1 (-51.1; -26.8)
Santa Catarina	55.1 (50.0-60.5)	38.9 (35.5-42.7)	40.2 (33.6-48.4)	-27 (-40.3; -10.8)
**Central-West**				
Goiás	80.3 (67.4-94.9)	60.8 (51.5-70.3)	62.3 (49.1-78.0)	-22.5 (-41.7; 1.9)
Mato Grosso	103.6 (76.5-129.0)	68.1 (61.8-75.3)	73.5 (60.5-88.2)	-29.1 (-47.5; 0.7)
Mato Grosso do Sul	106.3 (95.2-118.9)	89.9 (80.8-99.9)	74.4 (60.9-89.7)	-30 (-44.5; -13.4)
Distrito Federal	81.9 (70.9-94.5)	49.4 (44.6-55.0)	50.0 (40.7-60.6)	-38.9 (-52.3; -22.7)

**FIGURE 3:**
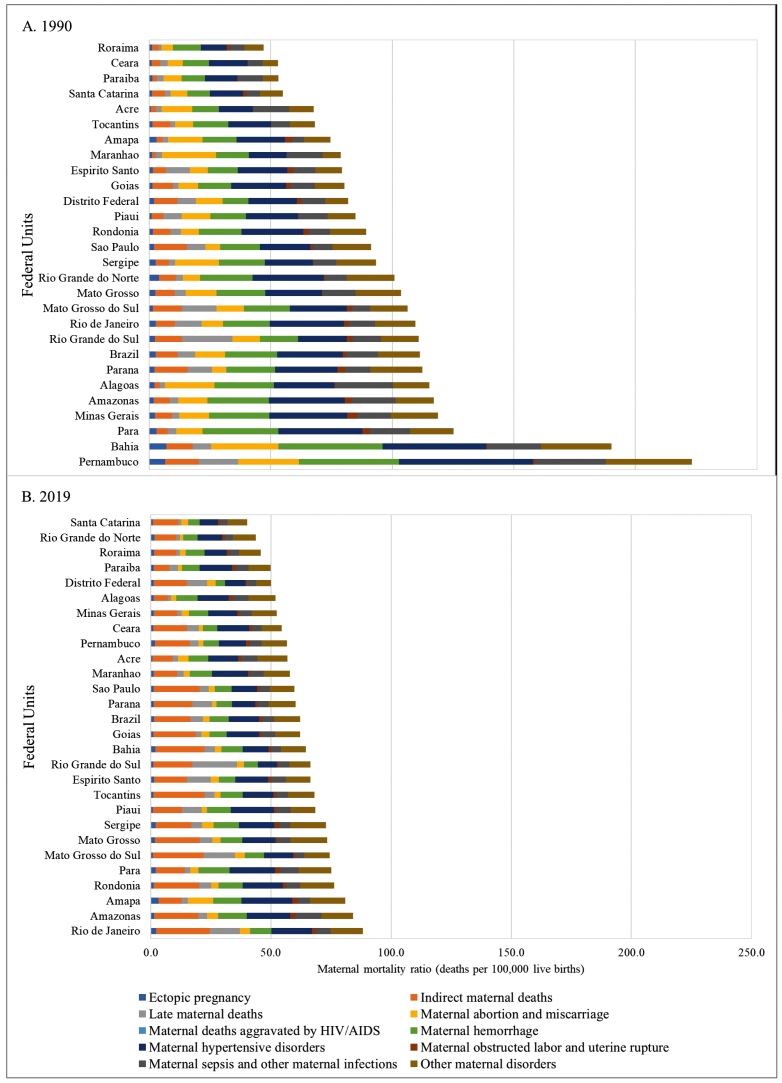
Maternal mortality ratio (MMR) Panel, women 10-54 years by state and death by specific-cause. GBD, 2019: **(A)** MMR by state and specific cause in 1990; **(B)** MMR by state and specific cause in 2019.

The MMR for specific causes of death and distributed by the FU is presented in Figure 3. In 1990 (Panel A), with the exception of Rio Grande do Sul, Maranhao, Acre, and Roraima, hypertensive disorders during pregnancy stood out as the main specific cause of death, and the MMR varied from 13.4 deaths per 100,000 live births in Paraiba to 55.1 deaths per 100,000 live births in Pernambuco. In Rio Grande do Sul, Maranhao, Acre, and Roraima, although the MMR for hypertensive disorders during pregnancy was high, other causes of death stood out. In Rio Grande do Sul, late maternal deaths were significant (MMR 20.8 /100,000 live births); in Maranhao, the abortions (MMR 22.3/100,000 live births). Deaths by Sepsis were high in Acre (MMR 14.6/100,000 live births), while deaths by hemorrhage were high in Roraima (MMR 11.5/100,000 live births).

The expressive reduction in MMR in 2019 (Panel B) as compared to 1990 (although with different degrees among the FU) caused a change in the profile of specific causes, with a higher occurrence of indirect maternal deaths and hypertensive disorders during pregnancy, followed by other maternal diseases. Rio Grande do Sul was the only state which continued to show high MMR for late maternal deaths (MMR 18.4/100,000 live births) when compared to 1990. Detailed estimates are shown in the Supplementary material Tables 7S **and** 8S **.**

**FIGURE 4:**
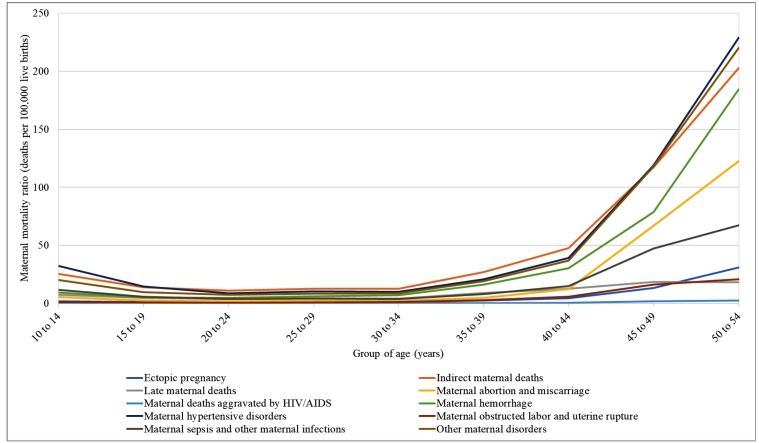
Maternal mortality ratio (MMR) by age group and cause-specific death in 2019. GBD, 2019.

Figure 4 shows the MMR by age groups and specific cause of death in 2019. It is relevant that the MMR is higher in the extreme age groups, and the risk is lower for the age group of 20 to 24 years. As age increases, the risk of maternal deaths increases exponentially and is 2.5-fold higher for those between 35 and 39 years of age, and 25-fold higher for those over 50 years of age, as compared to the age group of 20 to 24 years. The three main specific causes of death in each age group were indirect maternal deaths, hypertensive disorders during pregnancy, and other maternal disorders. As age increases, other causes gain importance, including hemorrhage (responsible for 35% of deaths) for women over 35 years of age, and abortion for those over 45 years of age (67.0 and 122.6 /100,000 live births for the age group of 45 to 49 years and for the age group of 50 to 54 years, respectively, accounting for nearly 11% of the maternal deaths. On the other hand, for the younger groups, after the three main causes, a high occurrence of maternal sepsis and of late maternal deaths was observed. MMR by age group and cause, with 95% UI, are shown in the Supplementary material Table 9S.

## DISCUSSION

Deaths associated with pregnancy and delivery have diminished in Brazil significantly since 1990, although the reduction falls short of the target of 75%, defined by the WHO (35.8/100,000 live births). In fact, one can notice that almost 100% of the decline occurred between 1990 and 2000, and remained stable in the following period. The national MMR is above the targets of the WHO and the SDG, and significant inequalities have been identified among the FU. In 2019, only three states showed estimates of less than 50 deaths per 100,000 live births. In more than 60% of the FU, indirect deaths are the biggest problem, especially for hypertensive disorders during pregnancy, which should be the focus of the efforts to reduce MMR in Brazil.

In 2015, only 11 countries reached the MDG in terms of reducing maternal deaths^9^, and in that year, Brazil was among the 30 countries which accounted for 80% of all maternal deaths in the world. Differently from global trends, in Brazil the biggest reduction of maternal deaths occurred between 1990 and 2001, with a 43% reduction. Meanwhile, in the rest of the world, the biggest reduction occurred between 2000 and 2017^20^. The decrease that happened in the beginning of the period can be explained partially by the efforts that took place before or immediately after 1990, such as the Integral Care Program for Women´s Health (PAISM, 1984), as well as other important primary care programs: Community Health Agent Program (1991) and Family Health Strategy (1994)^21^, which has improved access to health care.

From 2000 on, a series of other governmental strategies have been launched, aimed at improving health for women and children. These have had positive impacts on health for this segment of the population, including the Brazilian Ministry of Health's Program for Humanization of Prenatal and Childbirth Care (2000) and the National Pact for Reduction of Maternal and Neonatal Mortality (2004), which implemented actions to improve prenatal care, delivery, and postdelivery care. They also structured Committees for the Prevention of Maternal, Infant and Neonatal Mortality (CPMMIF, in Portuguese), throughout the country^22^, and created the Stork Network in 2011. These were important initiatives; however, the process of the investigation and recording of maternal deaths was improved in the country, which may account for the detection of more cases. In 2009, only 55% of female deaths were investigated in the country. From 2010 to 2018, the number increased from 77% to 91%^14^, which reveals improvement and, consequently, an increase in recorded cases. Improvements in maternal death vigilance might explain, at least partially, why the MMR became stable from 2010 onward. However, no detailed information of the fractions added to maternal surveillance was found to confirm this hypothesis.

In Brazil, the maternal deaths surveillance was regulated by Ordinance 1,119, signed on June 5th, 2008^23^, as one of the actions to curb maternal deaths. The Maternal Surveillance Secretariat instituted the investigation of deaths of women at a fertile age (occurring among women aged 1 to 49 years), excluding cases of death that happened 42 days after the end of pregnancy. The process of investigation is initiated by the Death Certificate (DC), and the conclusion of the epidemiological investigation is a responsibility of the Death Surveillance Reference Team from the city where the woman resides. Once the investigation is finished, the city of residence is responsible for entering the occurrence into the SIM system, within 120 days after the death. The deaths are registered by the Municipal Departments of Health, which are shared with the State Secretary and consolidated nationally by the Ministry of Health^23^.

However, there are still persistent inequalities and variations among the FU, which need to be addressed. It is necessary to evaluate the causes, the gaps in care and the structural problems of Brazil^24^ ^-^ ^27^. Widely known regional inequalities have been demonstrated by the GBD study. In 2019, the highest MMR was in Rio de Janeiro (90 maternal deaths per 100,000 live births), followed by the states of Amazonas and Amapa. There are practically no surveys about maternal mortality according to regions of the country, and when they do exist, they have significant rates of the underreporting of maternal deaths^28^.

Data from Rio de Janeiro had already identified a concentration of mortality in the poorest parts of the city, among young, uneducated women^29^. Other studies done in Brazil show differences related to ethnicity/color. When comparing white women to light-skinned and dark-skinned black women, the latter show worse indicators of prenatal adequation^30^. Furthermore, violence against women also represents an important maternal death factor^31^.

Between 1990 and 2019, the main cause of maternal deaths in Brazil were the hypertensive pregnancy diseases, with notable transition to mortality due to indirect causes, which include various conditions, from diabetes to infectious diseases. (Supplementary material Table 6S). The reduction of mortality by cardiovascular diseases and diabetes, observed in the Brazilian adult population^32^, does not seem to be reflected in the population of pregnant women. Several studies point to medical care failures in dealing with these diseases in pregnant women^33^ ^,^ ^34^. Studies show that, although the number of prenatal consultations is adequate, there is a lack of quality in the care provided, as well as in the implemented intervention measures^35^.

In addition to chronic diseases, the increase in infectious diseases during pregnancy has been documented in recent years. The rates of syphilis incidence in pregnant women have also increased in Brazil in recent years^36^ ^,^ ^37^, with great regional differences^38^. Studies show that the H1N1 pandemic showed an impact on the MMR, with an increase of 6% between 2008 and 2009^14^ ^,^ ^39^. Taking this number into consideration, the current scenario of a new pandemic again highlights a concern that pregnant women will be disproportionately affected^40^ ^,^ ^41^. The estimates of maternal mortality until mid-2020 had already indicated that Brazil was one of the countries with the highest maternal and post-partum mortality in the world^42^, reinforcing the inequalities that affect this segment of the population, which have not been addressed over the years.

Another important cause, which is difficult to measure and is not identified in the present study, refers to induced abortion. This practice has been performed over the years, and since it has not been decriminalized, the underreporting of causes resulting from bacterial infection related to induced abortion will persist. The risk of sepsis is also increased by other adverse events during pregnancy and delivery, such as spontaneous abortion, premature delivery, obstructed delivery or retained placenta after birth. This is aggravated further by the difficulties in access to medical care, especially for poorer and less educated women^43^ ^,^ ^44^.

The reduction in the MMR in Brazil and worldwide can be attributed to an expressive reduction in fertility rates, especially among younger women^20^. However, the change in the age pattern of fertility, with an increase in rates for older ages (when maternal risk is greater) might impact the MMR negatively. Moreover, we must consider that the reduction in fertility rates has not been homogeneous around the country. In low-income locations, especially in the North Region, and among less educated women, fertility is higher and more precocious^45^.

Advantages and disadvantages must be mentioned in terms of using the GBD estimates for maternal mortality in Brazil Among the advantages, one can highlight the methodology used by the HIME, which allows for a comparison of data and a correction of views. In addition to the solidity of the applied corrections, the GBD includes important and available sources.

Among the limitations of using secondary data to build estimates, which are already well-known, is the fact that this data is not available for the entire period. For the estimates of maternal deaths in Brazil, data up to 2017 was used; therefore, the final results for 2019 are based on recent history, data, and results of the chosen model. Moreover, the GBD study does not consider the different levels of investigation of maternal deaths in the country. No adjustments were done in terms of differences related to the proportion of deaths investigated and identified as maternal deaths. Such differences can partially explain the differences in the estimates published by the Ministry of Health and those published by the GBD^10^.

Finally, maternal mortality in Brazil did not reach the target established by the MDG and will most likely not reach the target established by the SDG, if the current trends prevail. Successful experiments to reduce MMR in the states and the municipalities of Brazil must be shared so that they can be adapted, thus allowing for the SDG targets to be reached by the country and in all of its geographic regions.

Among other factors, structural issues in medical care and in terms of the great social inequalities, as well as inequalities in terms of color/ethnicity, can explain the difficulties in reducing maternal deaths. The weakening of the Brazilian Unified Health System and the difficulties faced due to cuts in health budgets, because of Constitutional Amendment 95, as well as cuts in social programs, make the outlook of improvements in this indicator less than optimistic.
