# Supplementary material for: Maternal Mortality in Brazil, 1990 to 2019: a systematic analysis of the Global Burden of Disease Study 2019
Source: Rev Soc Bras Med Trop. 2022 Jan 28;55(Suppl 1):e0279-2021. doi: 10.1590/0037-8682-0279-2021 (PMC9009438; doi:10.1590/0037-8682-0279-2021)
Supplement: Supplementary file 1 [file 1678-9849-rsbmt-55-s01-e0279-2021-supp1.pdf]

TABLE 1S: Data sources used by Cause of death, by data source type and state. GBD, 2019.

	Number of sources	Number of years	Type of data source and coverage
**Brazil**	**51**	**849**	
Acre	33	32	Brazil Mortality Information System - Deaths 1985-2017
Alagoas	22	21	Brazil Mortality Information System - Deaths 1995,1997-2017
Amapa	38	37	Brazil Mortality Information System - Deaths 1980-2017
Amazonas	38	37	Brazil Mortality Information System - Deaths 1980-2017
Bahia	33	32	Brazil Mortality Information System - Deaths 1985-2017
Ceara	24	22	Brazil Mortality Information System - Deaths 1995-2017; Factors associated with maternal mortality among patients meeting criteria of severe maternal morbidity and near miss
Distrito Federal	38	37	Brazil Mortality Information System - Deaths 1980-2017
Espirito Santo	38	37	Brazil Mortality Information System - Deaths 1980-2017
Goias	38	37	Brazil Mortality Information System - Deaths 1980-2017
Maranhao	15	14	Brazil Mortality Information System - Deaths 2003-2017
Mato Grosso	33	32	Brazil Mortality Information System - Deaths 1985-2017
Mato Grosso do Sul	38	37	Brazil Mortality Information System - Deaths 1980-2017
Minas Gerais	38	37	Brazil Mortality Information System - Deaths 1980-2017
Para	38	37	Brazil Mortality Information System - Deaths 1980-2017
Paraiba	15	14	Brazil Mortality Information System - Deaths 2003-2017
Parana	38	37	Brazil Mortality Information System - Deaths 1980-2017
Pernambuco	30	27	Brazil Mortality Information System - Deaths 1990-2017; Maternal mortality in Pernambuco, Brazil: what has changed in ten years? ; [Epidemiological features of maternal deaths occurred in Recife, PE, Brazil (2000-2006)]
Piaui	25	24	Brazil Mortality Information System - Deaths 1993-2017; Incidence and determinants of severe maternal morbidity: a transversal study in a referral hospital in Teresina, Piaui, Brazil
Rio de Janeiro	39	37	Brazil Mortality Information System - Deaths 1980-2017; Maternal deaths in the city of Rio de Janeiro, Brazil, 2000-2003
Rio Grande do Norte	30	29	Brazil Mortality Information System - Deaths 1988-2017
Rio Grande do Sul	38	37	Brazil Mortality Information System - Deaths 1980-2017
Rondonia	37	36	Brazil Mortality Information System - Deaths 1981-2017
Roraima	38	37	Brazil Mortality Information System - Deaths 1980-2017
Santa Catarina	38	37	Brazil Mortality Information System - Deaths 1980-2017
Sao Paulo	42	37	Brazil Mortality Information System - Deaths 1980-2017; Applying the new concept of maternal near-miss in an intensive care unit; Maternal mortality due to arterial hypertension in São Paulo City (1995-1999); Maternal mortality in Campinas: evolution, under-registration and avoidance; [Mortality among women in reproductive age in the municipality of São Paulo, Brazil, 1986. II. Deaths by maternal causes]
Sergipe	25	24	Brazil Mortality Information System - Deaths 1992, 1994-2017
Tocantins	25	24	Brazil Mortality Information System - Deaths 1993-2017
