# Supplementary material for: Maternal Mortality in Brazil, 1990 to 2019: a systematic analysis of the Global Burden of Disease Study 2019
Source: Rev Soc Bras Med Trop. 2022 Jan 28;55(Suppl 1):e0279-2021. doi: 10.1590/0037-8682-0279-2021 (PMC9009438; doi:10.1590/0037-8682-0279-2021)
Supplement: Supplementary file 2 [file 1678-9849-rsbmt-55-s01-e0279-2021-supp2.pdf]

TABLE 2S: Number of data sources used by Mortality by state. GBD, 2019.

	Number of sources
**Brazil**	**126**
Acre	59
Alagoas	61
Amapa	59
Amazonas	60
Bahia	61
Ceara	61
Distrito Federal	60
Espirito Santo	60
Goias	60
Maranhao	61
Mato Grosso	60
Mato Grosso do Sul	60
Minas Gerais	60
Para	60
Paraiba	61
Parana	60
Pernambuco	61
Piaui	61
Rio de Janeiro	60
Rio Grande do Norte	61
Rio Grande do Sul	60
Rondonia	59
Roraima	59
Santa Catarina	60
Sao Paulo	59
Sergipe	61
Tocantins	49
