# Supplementary material for: Maternal Mortality in Brazil, 1990 to 2019: a systematic analysis of the Global Burden of Disease Study 2019
Source: Rev Soc Bras Med Trop. 2022 Jan 28;55(Suppl 1):e0279-2021. doi: 10.1590/0037-8682-0279-2021 (PMC9009438; doi:10.1590/0037-8682-0279-2021)
Supplement: Supplementary file 3 [file 1678-9849-rsbmt-55-s01-e0279-2021-supp3.pdf]

TABLE 3S: Covariates used for modeling maternal mortality. GBD, 2019.

Level	Covariate
Level 1	Age-specific fertility rate<br>Total fertility rate (log-transformed)<br>Maternal education (years per capita)<br>In-facility delivery (proportion)<br>Skilled birth attendance (proportion)<br>Neonatal mortality ratio (log-transformed)<br>Age-specific HIV mortality in females 10-54 (log-transformed)
Level 2	Antenatal care 1-visit coverage (proportion)<br>Antenatal care 4-visits coverage (proportion)<br>Age-standardised wasting (weight-for-height) summary exposure value (SEV)<br>Age-standardised stunting (height-for-age) SEV<br>Healthcare Access and Quality Index<br>Age- and sex-specific SEV for high body-mass index (BMI)<br>Age- and sex-specific SEV for high blood pressure (SBP)<br>Underweight women of reproductive age
Level 3	Socio-demographic Index<br>Mortality shock (cumulative rate in last 10 years)<br>LDI (log-transformed)<br>Hospital beds (per 1,000 population)
