# Supplementary material for: Maternal Mortality in Brazil, 1990 to 2019: a systematic analysis of the Global Burden of Disease Study 2019
Source: Rev Soc Bras Med Trop. 2022 Jan 28;55(Suppl 1):e0279-2021. doi: 10.1590/0037-8682-0279-2021 (PMC9009438; doi:10.1590/0037-8682-0279-2021)
Supplement: Supplementary file 4 [file 1678-9849-rsbmt-55-s01-e0279-2021-supp4.pdf]

TABLE 4S: Covariates included for modeling causes of individual death estimates. GBD, 2019.

Maternal subcause	Country-level covariates Direction
Maternal haemorrhage	In-facility delivery (proportion)<br>Skilled birth attendance (proportion)<br>Age- and sex-specific SEV for unsafe sanitation<br>Neonatal mortality ratio (log-transformed)<br>Maternal education<br>Healthcare Access and Quality Index
Maternal hypertensive disorders	Age- and sex-specific SEV for fasting plasma glucose (FPG)<br>Age- and sex-specific SEV for high body-mass index (BMI)<br>Age- and sex-specific SEV for high blood pressure (SBP)<br>Neonatal mortality ratio (log-transformed)<br>Hospital beds (per 1000 population)<br>Antenatal care 1-visit coverage (proportion)<br>Antenatal care 4-visits coverage (proportion)<br>Healthcare Access and Quality Index
Obstructed labour and uterine rupture	In-facility delivery (proportion)<br>Skilled birth attendance (proportion)<br>Underweight women of reproductive age<br>Neonatal mortality ratio (log-transformed)<br>Hospital beds (per 1000 population)<br>Age-standardised wasting (weight-for-height) SEV<br>Age-standardised stunting (height-for-age) SEV
Abortion and miscarriage	Abortion legality<br>Antenatal care 1-visit coverage (proportion)<br>Antenatal care 4-visits coverage (proportion)<br>Hospital beds (per 1,000 population)<br>Maternal education<br>Healthcare Access and Quality Index
Ectopic pregnancy	Abortion legality<br>Pelvic inflammatory disease age-standardised prevalence<br>Antenatal care 1-visit coverage (proportion)<br>Antenatal care 4-visits coverage (proportion)<br>Hospital beds (per 1,000 population)<br>Maternal education<br>Healthcare Access and Quality Index
Maternal sepsis and other maternal infections	In-facility delivery (proportion)<br>Skilled birth attendance (proportion)<br>Age- and sex-specific SEV for unsafe sanitation<br>Age- and sex-specific SEV for fasting plasma glucose (FPG)<br>Antenatal care 1-visit coverage (proportion)<br>Antenatal care 4-visits coverage (proportion)<br>LDI (log-transformed)<br>Healthcare Access and Quality Index
Other maternal deaths	In-facility delivery (proportion)<br>Skilled birth attendance (proportion)<br>Antenatal care 1-visit coverage (proportion)<br>Antenatal care 4-visits coverage (proportion)<br>LDI (log-transformed)<br>Age- and sex-specific SEV for high body-mass index (BMI)<br>Maternal education<br>Healthcare Access and Quality Index
Indirect maternal deaths	In-facility delivery (proportion)<br>Skilled birth attendance (proportion)<br>Antenatal care 1-visit coverage (proportion)<br>Antenatal care 4-visits coverage (proportion)<br>LDI (log-transformed)<br>Age- and sex-specific SEV for high body-mass index (BMI)<br>Maternal education<br>Healthcare Access and Quality Index
