# Supplementary material for: Maternal Mortality in Brazil, 1990 to 2019: a systematic analysis of the Global Burden of Disease Study 2019
Source: Rev Soc Bras Med Trop. 2022 Jan 28;55(Suppl 1):e0279-2021. doi: 10.1590/0037-8682-0279-2021 (PMC9009438; doi:10.1590/0037-8682-0279-2021)
Supplement: Supplementary file 5 [file 1678-9849-rsbmt-55-s01-e0279-2021-supp5.pdf]

TABLE 5S: Global Burden of Disease maternal disorders definitions.

Disorder	Definition	ICD-10	ICD-9
Maternal disorders	Maternal complications occurring during the pregnancy or within one year of the end of pregnancy for women aged 10 to 54 years.	C58-C58.0, N96, N98-N98.9, O00-O07.9, O09-O16.9, O20-O26.9, O28-O36.9, O40-O48.1, O60-O77.9, O80-O92.7, O96-O98.6, O98.8-O99.9	181-181.9, 630-636.9, 638-638.9, 640-679.1
Maternal hemorrhage	Any hemorrhage during pregnancy, of any volume, or postpartum hemorrhage with blood loss of at least 500 mL (just over two cups).	O20-O20.9, O43.2, O44-O46.9, O62-O62.9, O67-O67.9, O70, O72-O72.3	640-641.9, 661-661.9, 665, 666-666.9
Maternal sepsis and other maternal infections	Any infection, or complication from an infection, experienced by a mother.	O23-O23.9, O85-O86.8, O91-O91.2	659.3, 670-670.9
Maternal hypertensive disorders (includes severe pre-eclampsia and eclampsia)	High blood pressure during pregnancy in women who did not already have hypertension, or pre-eclampsia in women with pre-existing hypertension.	O10-O16.9	642-642.9
Obstructed labor and uterine rupture	Ruptured uterus, prolonged labor greater than 24 hours, baby's head is too large to travel through the pelvis, or baby is born in a breech position (bottom-first) requiring an intervention like a C-section.	O32-O33.9, O64-O66.9, O71-O71.9	652-653.9, 660-660.9, 665.0-665.3
Abortion and miscarriage	Induced abortion by a health care provider or as a complication of a miscarriage.	N96, O01-O07.9	630-632.9, 634-636.9, 638-638.9, 646.3
Ectopic pregnancy	When the fertilized egg implants somewhere other than the uterus, most commonly in the fallopian tubes.	O00-O00.9	633-633.9
Indirect maternal deaths	Deaths due to pre-existing conditions made worse by pregnancy.	O24-O25.3, O98-O98.6, O98.8-O99.9	646-646.2, 646.4-649.9
Late maternal deaths	Deaths from any cause that occur from six weeks to 12 months after pregnancy.	O96-O97.9	
Maternal deaths aggravated by HIV/AIDS	Deaths where the cause of death is pregnancy aggravated by HIV/AIDS.		
Other direct maternal disorders	All other direct maternal disorders, including anemia in pregnancy, gestational diabetes, and embolism.	C58-C58.0, N98-N98.9, O09-O09.9, O21-O22.9, O26-O26.9, O28-O31.8, O34-O36.9, O40-O43.1, O43.8-O43.9, O47-O48.1, O60-O61.9, O63-O63.9, O68-O69.9, O70.0-O70.9, O73-O77.9, O80-O84.9, O87-O90.9, O92-O92.7	181-181.9, 643-645.2, 650-651.9, 654-659.2, 659.4-659.9, 662-664.9, 665.4-665.9, 667-669.9, 671-679.1

Source: Institute for Health Metrics and Evaluation Client Services. Making the World a Healthier Place for Mothers: Trends and Opportunities for Action in Maternal Health. Seattle, WA: IHME, 2019.
