# Supplementary material for: Maternal Mortality in Brazil, 1990 to 2019: a systematic analysis of the Global Burden of Disease Study 2019
Source: Rev Soc Bras Med Trop. 2022 Jan 28;55(Suppl 1):e0279-2021. doi: 10.1590/0037-8682-0279-2021 (PMC9009438; doi:10.1590/0037-8682-0279-2021)
Supplement: Supplementary file 6 [file 1678-9849-rsbmt-55-s01-e0279-2021-supp6.pdf]

**TABLE 6S:** ICD-10 for indirect maternal deaths.

| Indirect maternal deaths | |
|---|---|
| O24-O25.3 | O24 Diabetes mellitus in pregnancy<br>O24.0 Pre-existing diabetes mellitus, insulin-dependent<br>O24.1 Pre-existing diabetes mellitus, non-insulin-dependent<br>O24.2 Pre-existing malnutrition-related diabetes mellitus<br>O24.3 Pre-existing diabetes mellitus, unspecified<br>O24.4 Diabetes mellitus arising in pregnancy<br>O24.9 Diabetes mellitus in pregnancy, unspecified<br>O25 Malnutrition in pregnancy |
| O98-O98.6 | O98 Maternal infectious and parasitic diseases classifiable elsewhere but complicating pregnancy, childbirth and the puerperium<br>O98.0 Tuberculosis complicating pregnancy, childbirth and the puerperium<br>O98.1 Syphilis complicating pregnancy, childbirth and the puerperium<br>O98.2 Gonorrhoea complicating pregnancy, childbirth and the puerperium<br>O98.3 Other infections with a predominantly sexual mode of transmission complicating pregnancy, childbirth and the puerperium<br>O98.4 Viral hepatitis complicating pregnancy, childbirth and the puerperium<br>O98.5 Other viral diseases complicating pregnancy, childbirth and the puerperium<br>O98.6 Protozoal diseases complicating pregnancy, childbirth and the puerperium |
| O98.8-O99.9 | O98.8 Other maternal infectious and parasitic diseases complicating pregnancy, childbirth and the puerperium<br>O98.9 Unspecified maternal infectious or parasitic disease complicating pregnancy, childbirth and the puerperium<br>O99 Other maternal diseases classifiable elsewhere but complicating pregnancy, childbirth and the puerperium<br>O99.0 Anaemia complicating pregnancy, childbirth and the puerperium<br>O99.1 Other diseases of the blood and blood-forming organs and certain disorders involving the immune mechanism complicating pregnancy, childbirth and the puerperium<br>O99.2 Endocrine, nutritional and metabolic diseases complicating pregnancy, childbirth and the puerperium<br>O99.3 Mental disorders and diseases of the nervous system complicating pregnancy, childbirth and the puerperium<br>O99.4 Diseases of the circulatory system complicating pregnancy, childbirth and the puerperium<br>O99.5 Diseases of the respiratory system complicating pregnancy, childbirth and the puerperium<br>O99.6 Diseases of the digestive system complicating pregnancy, childbirth and the puerperium<br>O99.7 Diseases of the skin and subcutaneous tissue complicating pregnancy, childbirth and the puerperium<br>O99.8 Other specified diseases and conditions complicating pregnancy, childbirth and the puerperium |
