# Supplementary material for: Maternal Mortality in Brazil, 1990 to 2019: a systematic analysis of the Global Burden of Disease Study 2019
Source: Rev Soc Bras Med Trop. 2022 Jan 28;55(Suppl 1):e0279-2021. doi: 10.1590/0037-8682-0279-2021 (PMC9009438; doi:10.1590/0037-8682-0279-2021)
Supplement: Supplementary file 7 [file 1678-9849-rsbmt-55-s01-e0279-2021-supp7.pdf]

TABLE 7S: Maternal mortality ratio (MMR), with 95% uncertainty interval, by age group (in years) and cause in 2019. GBD, 2019.

Maternal disorders	MMR, 95% uncertainty interval (UI)								
	10-14	15-19	20-24	25-29	30-34	35-39	40-44	45-49	50-54
Ectopic pregnancy	1.7(1.1;2.4)	1.1(0.7;1.6)	1.0(0.6;1.4)	1.2(0.8;1.7)	1.3(0.9;1.9)	2.6(1.7;3.8)	4.6(3.1;6.6)	13.4(8.8;19.2)	31.1(22.5;42.3)
Indirect maternal deaths	25.4(17.5;34.4)	13.8(9.6;18.8)	11.1(7.9;14.8)	12.6(9.2;16.8)	12.8(9.3;17.2)	27.1(19.4;35.9)	47.6(32.3;65.0)	117.5(81.6;160.4)	202.7(146.2;271.5)
Late maternal deaths	7.5(4.2;12.3)	4.7(3.1;7.2)	3.9(2.6;5.7)	4.4(3.1;6.0)	4.2(2.9;5.9)	8.9(6.3;12.5)	12.6(7.7;19.2)	18.6(12.9;27.4)	18.2(11.2;30.2)
Maternal abortion and miscarriage	5.5(2.8;9.6)	2.5(1.2;4.4)	1.8(0.9;3.2)	2.1(1.1;3.5)	2.1(1.0;3.6)	4.8(2.4;8.7)	12.3(6.3;21.0)	67.0(36.7;112.3)	122.6(72.8;185.3)
Maternal deaths aggravated by HIV/AIDS	0.0(0.0;0.0)	0.0(0.0;0.1)	0.0(0.0;0.1)	0.1(0.1;0.2)	0.1(0.1;0.2)	0.3(0.2;0.5)	0.6(0.4;0.9)	1.7(0.9;2.4)	2.5(1.4;3.6)
Maternal hemorrhage	9.5(5.6;14.4)	5.2(3.1;7.9)	4.7(2.8;7.1)	6.2(3.7;9.3)	7.1(4.2;10.9)	16.1(10.2;23.4)	30.2(18.9;45.7)	78.9(48.4;120.1)	184.6(124.1;257.8)
Maternal hypertensive disorders	32.3(25.5;39.7)	14.5(11.5;17.8)	8.7(6.9;10.7)	10.5(8.1;13.0)	10.1(8.0;12.6)	20.7(16.3;25.5)	39.2(31.0;49.0)	118.9(92.8;147.1)	229.0(186.1;283.6)
Maternal obstructed labor and uterine rupture	1.3(0.6;2.3)	0.7(0.4;1.3)	0.6(0.3;1.1)	0.9(0.5;1.6)	1.2(0.6;2.1)	2.9(1.4;5.0)	5.8(3.1;10.0)	16.2(8.7;28.0)	20.9(11.4;35.9)
Maternal sepsis and other maternal infections	11.6(7.1;17.9)	5.7(3.5;8.4)	3.9(2.5;5.8)	4.1(2.5;6.2)	3.7(2.2;5.8)	8.0(5.1;12.3)	14.9(9.4;22.7)	47.3(29.4;70.1)	67.4(45.2;96.0)
Other maternal disorders	20.2(12.9;31.4)	9.7(6.0;14.4)	7.5(4.8;11.0)	8.5(5.2;12.8)	8.8(5.5;13.1)	19.3(11.9;29.3)	36.8(23.6;55.7)	118.0(72.9;172.3)	220.1(143.1;310.7)
