# Supplementary material for: Maternal Mortality in Brazil, 1990 to 2019: a systematic analysis of the Global Burden of Disease Study 2019
Source: Rev Soc Bras Med Trop. 2022 Jan 28;55(Suppl 1):e0279-2021. doi: 10.1590/0037-8682-0279-2021 (PMC9009438; doi:10.1590/0037-8682-0279-2021)
Supplement: Supplementary file 8 [file 1678-9849-rsbmt-55-s01-e0279-2021-supp8.pdf]

TABLE 8S: Maternal mortality ratio (MMR) with 95% uncertainty interval by cause of death, women 10-54 years, Brazil and Federal Units, in 1990. GBD, 2019.

	MMR, 95% uncertainty interval (UI)									
**Year/Local**	**Ectopic pregnancy**	**Indirect maternal deaths**	**Late maternal deaths**	**Maternal abortion and miscarriage**	**Maternal deaths aggravated by HIV/AIDS**	**Maternal hemorrhage**	**Maternal hypertensive disorders**	**Maternal obstructed labor and uterine rupture**	**Maternal sepsis and other maternal infections**	**Other maternal disorders**
**1990**										
Acre	0.6(0.5;0.8)	2.3(1.7;3.1)	2.1(1.3;3.5)	12.6(8.6;17.4)	0(0;0)	10.9(7.9;14.4)	14.1(11.2;17.2)	0.2(0.1;0.3)	14.6(10.6;19.1)	10.2(7.2;13.6)
Alagoas	2.2(1.7;2.9)	2.2(1.6;2.9)	2.1(1;4.3)	20.3(14.7;27.5)	0(0;0)	24.4(18.4;31.4)	25.1(20.7;29.8)	0.3(0.2;0.5)	23.6(18.1;30)	14.9(11;19.8)
Amapá	3(2.2;4)	2.6(1.8;3.5)	2.2(0.8;4.9)	14.2(9.6;20)	0(0;0)	14(9.8;19.3)	19.8(14.8;24.7)	3.1(2;4.6)	4.7(3.2;6.8)	10.8(7.2;14.9)
Amazonas	1.9(1.4;2.6)	6.6(4.7;8.7)	3.5(1.8;6.1)	12(8;16.7)	0(0;0.1)	25.3(18.8;33.8)	31.2(24.3;37.8)	2.9(2;4.2)	18(13.1;23.9)	15.7(11.5;20.8)
Bahia	7.1(5.3;9.3)	10.8(8;14.3)	7.6(4.9;11.3)	27.8(19.2;38.7)	0(0;0.1)	42.7(31.9;55.7)	42.7(34.5;51.9)	0.5(0.3;0.8)	21.9(15.9;29)	29.1(21;39.1)
**Brazil**	**2.8(2.3;3.2)**	**8.9(7.6;10.4)**	**7.2(5.2;9.7)**	**12.4(9.7;15.1)**	**0(0;0.1)**	**21.4(18;25.2)**	**27(24.6;29.4)**	**1.5(1.2;2)**	**12.9(10.8;15.4)**	**17.3(14.4;20.5)**
Ceará	1(0.7;1.4)	3.6(2.6;4.9)	3(2;4.4)	6.2(4.2;8.8)	0(0;0)	10.8(7.7;14.7)	15.8(12.3;19.9)	0.1(0.1;0.2)	6.2(4.3;8.5)	6.1(4.3;8.5)
Distrito Federal	2(1.5;2.6)	9.7(7.4;12.6)	7.5(5.4;10.2)	11.1(7.7;15.2)	0(0;0.1)	10.5(7.7;13.9)	19.9(16.1;24)	1.9(1.2;2.8)	9.7(7.2;12.8)	9.5(7.1;12.7)
Espírito Santo	1.6(1.2;2.1)	5.4(4.1;6.8)	9.8(7.3;13.2)	7.5(5.3;10.2)	0(0;0)	12.3(9.3;15.7)	20.3(17.3;23.3)	2.7(1.8;3.8)	8.8(6.6;11.5)	11(8.5;14.3)
Goiás	1.3(1;1.7)	8.5(6.3;11.2)	2.1(0.9;4.2)	8.1(5.4;11.7)	0(0;0)	13.7(9.9;17.9)	22.6(18.1;27.8)	1.9(1.3;2.9)	9.8(7.2;13.2)	12.2(8.9;16.2)
Maranhão	1.2(0.8;1.6)	1.6(1.1;2.3)	2.4(1.5;3.8)	22.3(15.5;30.3)	0(0;0)	13.4(9.3;18.9)	15.5(11.5;20)	0.1(0;0.1)	14.8(10.5;20.3)	7.4(5;10.5)
Mato Grosso	2.5(1.7;3.5)	7.9(5.3;11)	4.5(2.7;7)	12.8(8.1;18.7)	0(0;0.1)	19.9(13.6;27.3)	23.4(16.6;30.8)	0.2(0.2;0.4)	13.5(9;19)	18.8(12.6;25.8)
Mato Grosso do Sul	1.5(1.2;1.9)	11.9(9;15.6)	14.4(10.4;19.4)	11.3(7.8;15.7)	0(0;0.1)	18.7(14.2;24.1)	23.5(19.5;27.5)	2.2(1.4;3.1)	7.5(5.4;10.1)	15.4(11.4;20)
Minas Gerais	2.4(1.9;2.9)	7.1(5.5;9)	2.9(1.3;5.8)	12.2(8.7;16.2)	0(0;0.1)	24.9(19.4;30.6)	32(27.5;36.4)	4(2.8;5.7)	13.9(10.7;17.8)	19.3(15.1;24.2)
Pará	3.1(2.3;4.1)	4.5(3.2;6.1)	3.5(1.8;6.2)	10.8(7.1;15.1)	0(0;0.1)	31.2(23.3;40.3)	34.6(27.4;42.7)	2.8(1.8;4.3)	16.7(11.9;22.2)	17.8(12.7;23.9)
Paraíba	1.3(1;1.7)	1.9(1.4;2.6)	2.7(1.8;4.1)	7.4(5.3;10)	0(0;0)	9.7(7.4;12.6)	13.4(11.2;16)	0.3(0.2;0.4)	10.1(7.6;12.9)	6.4(4.7;8.6)
Paraná	2.1(1.7;2.7)	13.8(11;17)	10(7.5;13.3)	5.9(4.1;8.5)	0(0;0.1)	19.9(15.3;24.9)	25.8(22.3;29.2)	3.2(2.2;4.3)	10.3(7.9;13.1)	21.5(16.6;27.2)
Pernambuco	6.7(5.2;8.4)	13.8(11;17.5)	16.3(11.9;21.9)	24.8(18;33.4)	0.1(0;0.1)	41.2(31.9;52.4)	55.1(47.9;62.9)	0.9(0.6;1.3)	29(22.6;36.2)	35.3(27.1;45)
Piauí	1.1(0.8;1.4)	4.8(3.7;6.3)	7.5(5.4;10.4)	11.7(8;16.1)	0(0;0)	14.7(11;19.5)	21.3(17.4;25.6)	0.1(0.1;0.2)	12(8.9;16)	11.6(8.4;15.1)
Rio de Janeiro	2.7(2.1;3.4)	8.1(6.4;10.1)	10.8(8.3;14.2)	8.8(6.2;12.1)	0.1(0;0.1)	19.1(15;24)	30.6(27.1;34.6)	2(1.4;2.8)	10.8(8.3;13.8)	16.5(12.9;20.9)
Rio Grande do Norte	4.1(3.1;5.4)	7(5.3;9.2)	2.7(1.3;5.4)	7.1(4.9;10.3)	0(0;0)	21.6(16.3;28.2)	29.4(24;35.9)	0.5(0.3;0.7)	8.7(6.3;11.7)	19.8(14.6;26.7)
Rio Grande do Sul	2.3(1.8;2.9)	11.2(9.1;14)	20.8(16;27.4)	11.3(7.9;15.3)	0(0;0.1)	15.6(12;19.7)	20.1(17.2;23)	2.2(1.6;3.1)	11.8(9;15.2)	15.6(11.9;20)
Rondônia	1.5(1;2.1)	7.3(4.7;10.3)	4.2(2.5;6.6)	7.3(4.4;10.9)	0(0;0.1)	17.6(11.4;23.9)	25.5(17.3;33.4)	2.3(1.4;3.4)	8.8(5.5;12.5)	14.7(9.3;20.6)
Roraima	1.3(0.8;1.8)	2.6(1.8;3.7)	1.2(0.5;2.2)	4.8(3;7.1)	0(0;0)	11.5(7.8;15.8)	10.6(7.4;13.7)	1.4(0.9;2.2)	5.8(3.8;8.2)	8.1(5.4;11.1)
São Paulo	2(1.6;2.5)	13.5(11.1;16.4)	7.6(4.5;11.1)	6.1(4.4;8.4)	0.1(0;0.1)	16.2(12.7;20.2)	20.8(18.1;24)	1.1(0.8;1.6)	7.9(6.1;10.3)	15.9(12.3;20)
Santa Catarina	1.2(0.9;1.6)	5.5(4.2;7)	2.1(0.6;5.5)	6.8(4.9;9.4)	0(0;0)	9.3(6.9;12.2)	13.6(11.5;16)	1.6(1.1;2.4)	5.3(4;7)	9.6(7.2;12.2)
Sergipe	2.7(2;3.6)	5.4(3.9;7.1)	2.6(1.4;4.7)	18(12.8;24.9)	0(0;0)	18.9(14;24.3)	19.6(15.9;23.6)	0.3(0.2;0.4)	9.5(6.8;12.6)	16.3(11.7;21.5)
Tocantins	1.4(1;1.8)	7.4(5.3;9.8)	1.8(0.8;3.5)	7.5(4.9;10.9)	0(0;0)	14.6(10.5;19.5)	17.6(13.6;21.9)	0.2(0.1;0.3)	7.5(5.4;10.1)	10.3(7.2;14.1)
