# Supplementary material for: Maternal Mortality in Brazil, 1990 to 2019: a systematic analysis of the Global Burden of Disease Study 2019
Source: Rev Soc Bras Med Trop. 2022 Jan 28;55(Suppl 1):e0279-2021. doi: 10.1590/0037-8682-0279-2021 (PMC9009438; doi:10.1590/0037-8682-0279-2021)
Supplement: Supplementary file 9 [file 1678-9849-rsbmt-55-s01-e0279-2021-supp9.pdf]

TABLE 9S: Maternal mortality ratio (MMR) by cause of death, women 10-54 years, Brazil and Federal Units, in 2019. GBD, 2019.

Year/Local	MMR, 95% uncertainty interval (UI)									
	Ectopic pregnancy	Indirect maternal deaths	Late maternal deaths	Maternal abortion and miscarriage	Maternal deaths aggravated by HIV/AIDS	Maternal hemorrhage	Maternal hypertensive disorders	Maternal obstructed labor and uterine rupture	Maternal sepsis and other maternal infections	Other maternal disorders
**2019**										
Acre	0.7(0.5;0.9)	8.4(6.3;10.8)	2.2(1.2;3.8)	4.5(3;6.4)	0.1(0;0.1)	8.1(5.8;10.9)	12.5(10.3;15)	1.4(0.9;2.1)	6.6(4.9;8.7)	12.5(9.2;16.4)
Alagoas	1.2(0.9;1.6)	5.9(4.2;7.8)	1.3(0.6;2.6)	2.2(1.4;3.2)	0.1(0;0.1)	8.9(6.3;11.9)	13(10.1;16.1)	1.7(1.1;2.5)	6.4(4.6;8.6)	11.3(8;15.2)
Amapá	3.4(2.5;4.4)	9.6(7.2;12.7)	2.4(1;5.5)	10.6(7.2;14.5)	0.2(0.1;0.3)	11.7(8.5;15.8)	21.1(17.1;25.7)	2.4(1.5;3.4)	5.1(3.7;6.8)	14.7(10.9;19.2)
Amazonas	1.4(1.1;1.9)	18.4(13.5;24)	3.6(2.1;5.9)	4.5(2.9;6.6)	0.3(0.1;0.4)	11.7(8.3;15.7)	18.2(14.5;22.8)	2.1(1.4;3.2)	10.8(7.9;14.4)	13.2(9.5;17.7)
Bahia	1.9(1.3;2.5)	20.6(15.1;27)	4.2(2.9;6)	2.7(1.7;4.1)	0.1(0.1;0.2)	8.8(6;12.5)	10.7(7.9;13.9)	1.3(0.8;2)	3.9(2.7;5.5)	10.4(7.2;14.5)
**Brazil**	**1.4(1.2;1.7)**	**15.2(13.1;17.7)**	**5(3.9;6.4)**	**2.8(2.1;3.6)**	**0.1(0.1;0.2)**	**7.8(6.4;9.5)**	**12.7(11.4;14)**	**1.3(1;1.6)**	**5(4.2;6)**	**10.7(8.9;13)**
Ceará	1.1(0.7;1.5)	14(10;18.9)	4.9(3.2;7)	1.7(1;2.5)	0.1(0;0.2)	5.9(4;8.6)	13.3(9.7;17.5)	1.4(0.8;2.1)	3.7(2.4;5.3)	8.5(5.7;12.1)
Distrito Federal	1.3(0.9;1.7)	13.9(10.6;18.2)	8.2(5.7;11.4)	3.7(2.4;5.3)	0.1(0;0.1)	3.7(2.6;5.4)	8.6(6.7;11)	0.5(0.3;0.8)	3.8(2.7;5.2)	6.2(4.4;8.5)
Espírito Santo	1.3(1;1.8)	13.8(10.2;17.9)	9.8(7.2;13.2)	3.5(2.2;5.2)	0.2(0.1;0.3)	6.7(4.5;9.2)	13.5(10.4;16.9)	1.4(0.9;2.1)	6(4.3;8)	10.3(7.5;14.3)
Goiás	1.1(0.8;1.5)	17.8(13.1;23.3)	2.2(1;4.3)	3.4(2.2;5)	0.1(0;0.2)	7.1(4.8;10)	13.5(10.3;17.2)	1(0.6;1.6)	5.7(3.9;8)	10.4(7.2;14.8)
Maranhão	1.2(0.9;1.8)	9.8(6.7;13.6)	2.7(1.7;4.2)	2.5(1.6;3.8)	0.1(0.1;0.2)	9.1(6.1;12.8)	14.9(10.8;19.8)	1(0.6;1.6)	5.6(3.8;7.8)	11(7.4;15.6)
Mato Grosso	1.7(1.2;2.3)	18.7(14.1;24.3)	5.1(3.3;8)	3.6(2.4;5.3)	0.2(0.1;0.3)	8.8(6.2;12.4)	13.9(10.8;17.3)	0.8(0.5;1.2)	5.3(3.8;7.2)	15.4(11.3;21)
Mato Grosso do Sul	1(0.7;1.3)	21.2(15.9;27.4)	13(9.2;17.5)	4(2.6;5.9)	0.1(0.1;0.2)	8(5.7;11.3)	12.1(9.5;15.3)	0.9(0.6;1.3)	3.7(2.6;5.1)	10.5(7.6;14.4)
Minas Gerais	1.2(0.9;1.6)	10(7.4;13)	1.7(1;2.7)	3(2;4.4)	0.1(0;0.1)	8(5.7;10.8)	12.1(9.6;15)	1.1(0.7;1.6)	4.9(3.5;6.6)	10.5(7.5;14.1)
Pará	2.2(1.6;2.9)	12.1(9;15.8)	2.2(1.3;3.5)	3.4(2.3;4.9)	0.2(0.1;0.3)	12.9(9.3;17.1)	18.9(15.3;23)	2.2(1.5;3.3)	7.6(5.7;10.4)	13.6(9.8;18.4)
Paraíba	1.3(0.9;1.7)	6.5(4.8;8.6)	3.4(2.2;5.2)	1.8(1.2;2.6)	0.1(0;0.1)	7.2(5.1;9.8)	13.6(10.8;17.1)	1.5(1;2.3)	5.3(3.7;7.2)	9.1(6.5;12.3)
Paraná	1.2(0.8;1.6)	16.1(12.4;20.1)	8.3(6.3;10.9)	1.9(1.2;2.8)	0.1(0.1;0.2)	6.3(4.6;8.6)	9.7(7.6;12.2)	1(0.6;1.5)	4.5(3.2;6.2)	11.2(8.3;15)
Pernambuco	1.8(1.3;2.3)	14.5(10.9;18.8)	3.5(2.4;5.2)	2.2(1.4;3.2)	0.1(0.1;0.2)	6.3(4.5;8.6)	11.3(8.9;13.9)	1.7(1.1;2.5)	4.8(3.4;6.4)	10.5(7.6;14)
Piauí	1(0.7;1.3)	12(9;15.9)	8.2(6.1;10.9)	2.3(1.5;3.4)	0.1(0.1;0.2)	9.8(7.1;13.3)	17.8(14.1;21.9)	1.2(0.7;1.8)	5.9(4.3;8)	10.2(7.4;13.7)
Rio de Janeiro	2.2(1.7;2.9)	22.5(17.2;28.4)	12.3(8.7;16.3)	4.4(2.8;6.3)	0.4(0.2;0.6)	8.5(6;11.8)	16.9(13.7;20.8)	1.6(1;2.3)	6.1(4.5;8.2)	13.3(9.9;17.7)
Rio Grande do Norte	1.5(1.1;2.2)	9.1(6.4;12.4)	1.5(0.7;2.8)	1.4(0.9;2)	0.1(0;0.1)	5.8(4;8.2)	10.3(7.5;13.6)	1.1(0.7;1.8)	3.3(2.2;4.5)	9.6(6.6;13.8)
Rio Grande do Sul	1.1(0.8;1.4)	16.3(12.3;21.1)	18.4(14.3;23.4)	3.1(2.1;4.4)	0.3(0.2;0.5)	5.4(3.7;7.5)	8(6.2;10)	0.7(0.4;1.1)	4.5(3.2;6.4)	8.6(6.2;11.8)
Rondônia	1.2(0.9;1.7)	18.9(14;24.3)	5.1(3.2;7.8)	3.1(2;4.5)	0.2(0.1;0.3)	9.9(6.8;13.6)	16.6(13.3;20.7)	1.4(0.9;2)	5.8(4.1;7.7)	14.2(10.1;19.5)
Roraima	1.4(1;1.8)	9.2(7;11.9)	1.5(0.6;3.4)	2.5(1.7;3.6)	0.1(0.1;0.2)	7.7(5.6;10.3)	9.3(7.4;11.4)	0.9(0.6;1.3)	4.1(3;5.6)	9(6.5;12.1)
São Paulo	1.3(1;1.7)	19(14.8;24.1)	3.8(2.8;5.2)	2.5(1.7;3.6)	0.1(0;0.2)	6.8(4.9;9.3)	10.7(8.7;13.1)	0.9(0.6;1.3)	4.3(3.1;5.8)	10.3(7.6;13.8)
Santa Catarina	0.9(0.6;1.2)	10.7(8.1;13.6)	1.2(0.5;2.8)	2.9(1.9;4.1)	0.1(0.1;0.2)	4.6(3.3;6.4)	7.7(6.1;9.6)	0.9(0.6;1.3)	3.1(2.3;4.4)	8.2(6;10.7)
Sergipe	2.1(1.5;2.9)	14.9(10.7;19.8)	4.5(2.2;8.2)	4.7(3;7)	0.1(0.1;0.2)	10.6(7.3;15)	14.6(11;18.7)	2(1.2;3.1)	4.6(3.2;6.4)	14.9(10.4;20.7)
Tocantins	1.1(0.8;1.5)	21.3(15.8;28)	4(1.9;7.2)	2.6(1.7;3.7)	0.1(0.1;0.2)	9(6.2;12.7)	12.8(9.8;16.5)	1.2(0.8;1.8)	4.8(3.4;6.7)	11(7.8;15.3)
